# Quick diagnosis, staging, and treatment of HCV infection among people living in prison: Opinion expert panel

**DOI:** 10.3389/fpubh.2022.926414

**Published:** 2022-10-04

**Authors:** Vito Fiore, Giuseppe De Matteis, Emanuele Pontali, Andrea De Vito, Sandro Panese, Nicholas Geremia, Ivana Maida, Stefania Artioli, Giulio Starnini, Giordano Madeddu, Sergio Babudieri

**Affiliations:** ^1^Unit of Infectious Diseases, Department of Medical, Surgical and Experimental Sciences, University of Sassari, Sassari, Italy; ^2^Health Protection for Adults and Youth Unit, Penitentiary Institute, Salerno, Italy; ^3^Infectious Disease Unit, Galliera Hospital, Genoa, Italy; ^4^Unit of Infectious Diseases, Department of Clinical Medicine, Dell'Angelo Hospital, Venice, Italy; ^5^Infectious Diseases and Hepatology Unit, Sant'Andrea Hospital La Spezia, La Spezia, Italy; ^6^Medicina Protetta-Unit of Infectious Diseases, Belcolle Hospital, Viterbo, Italy

**Keywords:** prison health, health inequalities, epidemiology, healthcare, vulnerable groups

## Introduction

Hepatitis C virus (HCV) infection prevalence among people living in prison (PLiP) is higher than in the general population (1). Because of high-risk behaviors and the probability of HCV transmission in the community after release, the prison population is considered one of the most important targets for HCV micro-elimination strategies.

As of March 2022, 54,609 PLiP were present in the 190 Italian penitentiary institutes, according to Ministry of Justice data (2).

According to the most recent literature, HCV seroprevalence among PLiP in Italy is up to 14.05% (3, 4), with active HCV infections in about 41% of cases (5).

As a consequence, it is estimated that more than 7,000 cases of HCV antibody positivity with more than 3,000 cases of active HCV infections may exist in Italian penitentiary settings.

Because of this, the Italian Ministry of Health funded the introduction of free HCV screening for identification among specific subpopulations, such as PLiP and people who inject drugs (PWIDs). The target is HCV elimination, according to the World Health Organization (WHO)'s 2030 global elimination targets (6), with a quick linkage to care in these difficult settings (7).

In the direct-acting antivirals (DAAs) era, the short schedules and the low risk of adverse reactions increased the number of treatments among infected patients. For these reasons, numerous papers have been published on the feasibility and efficacy of HCV therapy in prison settings. Furthermore, literature on updates in HCV epidemiology and the cascade of care in penitentiary settings has been rising in the last few years.

## Expert panel

The Italian Society of Medicine and Penitentiary Health (SIMSPe) invited a panel of experts on HCV management of HCV from among the most active operators in the field of health within Italian prisons. The experts were involved in meetings organized to re-evaluate the most recent literature, discuss their clinical experience, and define new practical recommendations for the approach to HCV micro-elimination in the prison settings.

## Prison settings specificity

The prison population has a different risk profile compared with the outside community. High-risk sexual behaviors, injection drug use, and unsterile tattooing are widely present among PLiP (8, 9). Furthermore, incarcerated people are not a stable population but have a high turnover (around 40%/year).

Of note, over 30% of incarcerations in Italy are due to drug-related crimes, with a high prevalence of PWIDs (2). Moreover, numerous comorbidities are present, such as a wide diffusion of psychiatric disorders (10).

There are no clear reports about barriers to achieving HCV treatment for PLiP in the DAAs era. However, the high drug costs, combined with the uncertainty of being able to complete the treatment, may be reasons against the choice to start treatment in the prison population (11).

For example, when considering PWIDs, the literature reports screening coverage of <70% in methadone clinics, and low rates (<10%) of evaluation for HCV treatment (12–14).

As a consequence, despite the low median age, social and legal reasons make PLiP one of the most underserved populations in terms of healthcare provision and use (15–17).

## Hepatitis C virus epidemiology in prison settings

According to the literature, HCV prevalence in penitentiary settings is in continuous evolution. One of the most important studies carried out in 2005 reported an HCV seroprevalence of 38% (18). Then, the epidemiology drastically changed in the last 5 years, with an HCV antibody prevalence of <20% in the penitentiary institutes. In 2016, Foschi et al. documented an HCV seroprevalence of 9.8% among PLiP (3).

Masarone et al. reported data on 670 PLiP, reporting an HCV seroprevalence of about14% (4).

The most recent multicenter study on HCV testing and treatment models in prison settings, including above 2,500 PLiP, showed HCV seroprevalence of about 10%. Active infection was reported in up to 44% of cases. PWIDs represented 66% of active infections (5).

Substantially, the DAAs availability seems to have already had an impact on HCV epidemiology. However, the seroprevalence is still three times higher than the national figure (19).

## Pre-counseling advantages

Despite the lack of data on tailored interventions to increase the HCV cascade of care in prison settings (20), a recent Italian study highlighted high rates of screening acceptance, fast screening, staging, and retention in care while applying an approach based on specific educational programs before the screening offer (5). As a consequence, a pre-counseling phase is highly recommended before starting screenings in the prison population.

## Quick tests for HCV screening

In the last years, the screening for HCV using quick tests has been highlighted as a major opportunity to implement point-of-care (POC) HCV testing in prison settings. This method showed a high diagnostic performance, with the advantage of a rapid uptake in how many patients can be seen (21). Two recent national studies highlighted the high patient uptake with this diagnostic procedure (4, 5). Furthermore, POC testing allows a rapid step-by-step path with the possibility of a fast-track treatment for viremic patients. Nowadays, not all penitentiary institutes are homogeneous in POC testing for HCV. This document proposes to implement the use of HCV quick tests, making it a standard procedure in penitentiary settings.

## Quick HCV-RNA testing: Lights and shadows

As previously discussed, a quick micro-elimination strategy seems to be the best strategy when approaching patients living with HCV, particularly in challenging settings. As reported by the literature, there is a high prevalence of undiagnosed chronic HCV infections (22–24). In this field, the need to overcome barriers, such as phlebotomy and sample transport in adequately equipped laboratories has been highlighted (25). Regarding this, HCV-RNA rapid testing has been discussed as an advantage in removing HCV elimination obstacles (25). In 2021, Izzo et al. reported data on 22 new PLiP in the period between January and February of 2020. Overall, 62 (50.8%) subjects underwent HCV-RNA quick testing through blood sampling, of which four (6.4%) subjects were found to be HCV-RNA positive. None of the HCV-active PLiP were lost to follow-up between HCV-RNA detection and treatment proposal. The use of a high-speed test-and-treat protocol for HCV infection was demonstrated to be effective in avoiding the number of patients lost to follow-up in HCV-positive new PLiP during the period between detection and treatment (26). Other methodologies for rapid diagnosis are possible, according to different strategies that involve different operating procedures, such as reflex test HCV-Ab on venous (serological test is performed for HCV-Ab and, in case of positivity, the laboratory will immediately carry out, on the same sample, the detection of HCV-RNA), rapid test for HCV Ab, and rapid test for HCV-RNA with blood by a capillary puncture. However, HCV genotyping is still required, and is strongly recommended by the national guidelines (27). Furthermore, phlebotomy is still required for blood tests, despite the availability of indirect staging methods. For this reason, a test-and-treat approach without phlebotomy among PLiP is not recommended by this opinion expert panel.

## Staging and follow-up

The advantage of fibrosis staging with FIB-4 value and APRI score in difficult settings has been well demonstrated (5). This would allow to start treatments without delays for PLiP. Furthermore, if platelet count is <100 × 10^9^/L, international normalized ratio (INR) should be obtained in the pre-treatment phase. HCV-RNA should be tested 4 weeks after therapy initiation, and 12 weeks after therapy completion to demonstrate the SVR12 (28).

The use of a score staging system and short follow-up schedules in prison settings is highly recommended by this expert panel.

## Retention in care

International data highlighted the high influence of prison release and transfer on retention in care and sustained virologic response (SVR) achievement in prison settings (29). The majority of studies showed SVR12 rates of about 90% among people retained in follow-up. However, PLiP achieving SVR on the intention-to-treat analysis is reported as about 70% due to loss to follow-up (30–32).

Nevertheless, the recent national data support a linkage to care approach. Operationally, patients next to release or transfer should not immediately start DAAs but should be staged and linked to care in the new penitentiary institute or in the referral territorial service (5). This would avoid unplanned treatment interruptions, resulting in a higher treatment coverage and SVR12. For this reason, staging and linkage to care with the specialist support are highly recommended by this expert panel.

## Micro-elimination model proposed by the Italian society of medicine and penitentiary health

**Figure 1:**
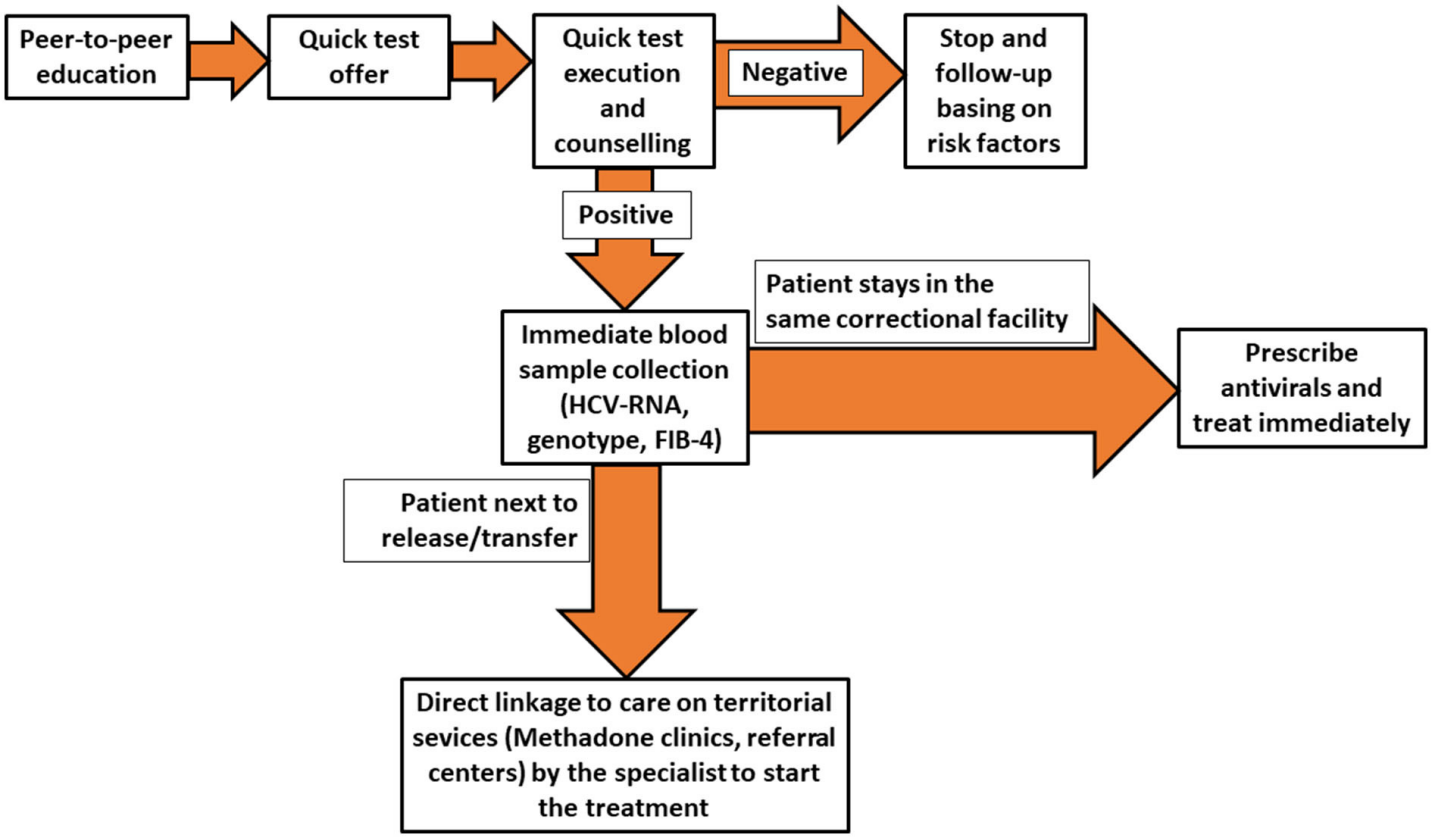
Flow-chart showing the multidisciplinary approach to Hepatitis C virus (HCV) diagnosis and treatment in prison settings promoted by the Italian Society of Medicine and Penitentiary Health.

The HCV micro-elimination model in prison promoted by SIMSPe is based on a multidisciplinary approach, with different professional involvement, with the aim to maximize the HCV screening provision and use among PLiP. The principal objective is to identify those who ignore their HCV status, test them for HCV, and promote the linkage-to-care of viremic HCV patients in detention centers and if necessary in territorial services. The micro-elimination model has been reported in Figure 1.

## Final remarks

Dedicated and nationally homogeneous interventions should be applied in penitentiary settings. The actual funding promoted by the Italian Ministry of Health would allow to implement rapid screening among hard-to-reach populations, such as incarcerated patients. A multidisciplinary model based on prison operators', nurses', and physicians' cooperation represents a successful approach to HCV test-staging-and-treatment in Italian prison settings.

## Author contributions

All authors listed have made a substantial, direct, and intellectual contribution to the work and approved it for publication.

## Conflict of interest

The authors declare that the research was conducted in the absence of any commercial or financial relationships that could be construed as a potential conflict of interest.

## Publisher's note

All claims expressed in this article are solely those of the authors and do not necessarily represent those of their affiliated organizations, or those of the publisher, the editors and the reviewers. Any product that may be evaluated in this article, or claim that may be made by its manufacturer, is not guaranteed or endorsed by the publisher.
